# Peripheral nerve injury and TRPV1-expressing primary afferent C-fibers cause opening of the blood-brain barrier

**DOI:** 10.1186/1744-8069-6-74

**Published:** 2010-11-02

**Authors:** Simon Beggs, Xue Jun Liu, Chun Kwan, Michael W Salter

**Affiliations:** 1 Program in Neurosciences & Mental Health, Hospital for Sick Children, Department of Physiology, University of Toronto, and University of Toronto Centre for the Study of Pain, Toronto, ON, Canada

## Abstract

**Background:**

The blood-brain barrier (BBB) plays the crucial role of limiting exposure of the central nervous system (CNS) to damaging molecules and cells. Dysfunction of the BBB is critical in a broad range of CNS disorders including neurodegeneration, inflammatory or traumatic injury to the CNS, and stroke. In peripheral tissues, the vascular-tissue permeability is normally greater than BBB permeability, but vascular leakage can be induced by efferent discharge activity in primary sensory neurons leading to plasma extravasation into the extravascular space. Whether discharge activity of sensory afferents entering the CNS may open the BBB or blood-spinal cord barrier (BSCB) remains an open question.

**Results:**

Here we show that peripheral nerve injury (PNI) produced by either sciatic nerve constriction or transecting two of its main branches causes an increase in BSCB permeability, as assessed by using Evans Blue dye or horseradish peroxidase. The increase in BSCB permeability was not observed 6 hours after the PNI but was apparent 24 hours after the injury. The increase in BSCB permeability was transient, peaking about 24-48 hrs after PNI with BSCB integrity returning to normal levels by 7 days. The increase in BSCB permeability was prevented by administering the local anaesthetic lidocaine at the site of the nerve injury. BSCB permeability was also increased 24 hours after electrical stimulation of the sciatic nerve at intensity sufficient to activate C-fibers, but not when A-fibers only were activated. Likewise, BSCB permeability increased following application of capsaicin to the nerve. The increase in permeability caused by C-fiber stimulation or by PNI was not anatomically limited to the site of central termination of primary afferents from the sciatic nerve in the lumbar cord, but rather extended throughout the spinal cord and into the brain.

**Conclusions:**

We have discovered that injury to a peripheral nerve and electrical stimulation of C-fibers each cause an increase in the permeability of the BSCB and the BBB. The increase in permeability is delayed in onset, peaks at about 24 hours and is dependent upon action potential propagation. As the increase is mimicked by applying capsaicin to the nerve, the most parsimonious explanation for our findings is that the increase in permeability is mediated by activation of TRPV1-expressing primary sensory neurons. Our findings may be relevant to the development of pain and neuroplastic changes in the CNS following nerve injury. In addition, our findings may provide the basis for developing methods to purposefully open the BBB when needed to increase brain penetration of therapeutic agents that might normally be excluded by an intact BBB.

## Background

The blood-brain barrier (BBB) is a highly specialized structure crucial for the maintenance of central nervous system (CNS) homeostasis [1,2]. The basis of the barrier in the brain, and the corresponding barrier in the spinal cord - the blood-spinal cord barrier (BSCB), is a network of endothelial cells joined by tight junctions that line the blood vessels within the CNS [3,4]. The core 'neurovascular unit' comprises endothelial cells, pericytes and astrocytic endfeet embedded within their basal laminae. The space between the astrocytic endfeet, which make up the abluminal surface of CNS capillaries, and the endothelial cells/pericytes represents the interface between the blood and CNS. The BBB is highly restrictive with only a subset of small molecular weight, diffusible molecules readily crossing from the blood into the CNS parenchyma. Thus, most substances are normally precluded from entering the CNS by the BBB. However, in many CNS pathological states the BBB becomes disrupted, allowing entry of substances from blood into the CNS, and this disruption is considered a key step for disorders such as traumatic injury, stroke and neurodegeneration.

In peripheral tissues, vascular permeability is normally greater than in the CNS, although there is a vascular-tissue barrier that excludes, for example, large proteins from entering the tissue. It has long been known that peripheral vascular permeability in skin and other tissues can be markedly increased by antidromic discharges in primary sensory neurons, allowing large proteins to leak through capillaries thereby causing plasma extravasation [5-7]. This plasma extravasation, together with the vasodilation that is produced by sensory nerve stimulation, comprise neurogenic inflammation which is mediated by the release of the peptides substance P and calcitonin gene-related peptide (CGRP) from peripheral terminals of peptidergic C-fibers [6,8,9]. In accordance with Dale's Principle [10], discharge activity in peptidergic C-fibers also releases substance P and CGRP from the central terminals of primary afferents in the spinal cord dorsal horn [11,12]. However, it has been found that vascular permeability in the dorsal horn is not increased by activating C-fibers, at least over the time course of peripheral neurogenic inflammation [13]. Therefore, it has been assumed that although activity in sensory nerves causes rapid increases in vascular permeability in peripheral tissues, this activity is not capable of causing vascular permeability to increase in the CNS.

Discharge activity in primary afferents, particularly that initiated by injury to sensory nerves, may have slowly developing and persistent consequences in the nervous system that can lead to chronic pain states [14]. Emerging evidence indicates that one such consequence of peripheral nerve injury (PNI) is the entry of monocytes [15] and T cells [16,17] from the circulation into the spinal dorsal horn. Because the accumulation of these normally circulating cells is apparent many hours or days after the nerve injury, we wondered whether the BSCB may be disrupted at these times, as has been suggested at longer times after spinal nerve transection [18]. Therefore, here we investigated whether PNI or activation of primary afferent C-fibers may cause increased vascular permeability in the CNS but over a time course beyond that of neurogenic inflammation. Preliminary results of portions of this work have been reported [19].

## Results

### Peripheral nerve injury causes delayed opening of the blood-spinal cord barrier

#### BSCB permeability is increased by chronic constriction injury (CCI)

**Figure 1:**
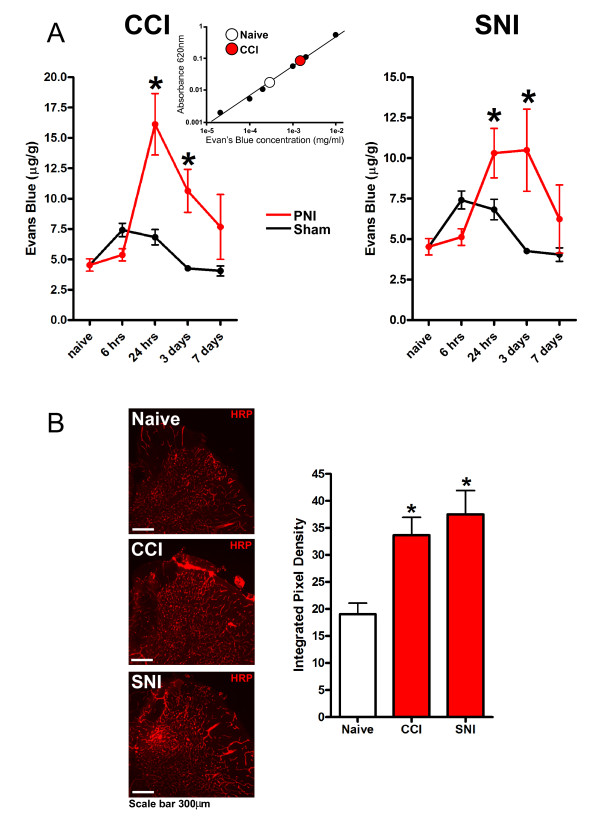
***Peripheral nerve injury (PNI) increases BSCB permeability***. A: Increased permeability to Evans Blue of spinal cord from naïve rats, sham operated rats or rats with chronic constriction injury (CCI, left) or spared nerve injury (SNI, right). Inset: Standard curve of Evans blue and the representative reading of naïve rats or CCI rats. B: Increased permeability to horseradish peroxidase (HRP) following PNI. Left, representative spinal cord sections of HRP extravasation 24 hours after SNI, CCI or naïve rats. Right, histogram showing the integrated pixel density of HRP signaling. Data are presented as mean ± SEM; *p < 0.05 compared to naïve; n = 4-6 per group. All measurements are from lumbar spinal cord ipsilateral to PNI stimulation.

We assessed the integrity of the BSCB with intravenously administered Evans Blue dye. Evans Blue binds to albumin in the circulation producing a molecular complex of sufficient molecular size that it does not cross the intact BSCB or BBB. Thus, there is minimal accumulation of Evans Blue in the spinal cord, or brain, under basal conditions in naïve animals (Figure 1). To investigate whether BSCB permeability might be altered by CCI we administered Evans Blue at varying times after the nerve injury, induced by placing a polythene cuff around the nerve, and quantified the amount of Evans Blue in the dorsal spinal cord ipsilateral to the injury. We found that 6 hours after the CCI surgery, the level of Evans Blue accumulating was not different from that of the dorsal horn of naïve animals, or animals that we subjected to sham surgery (Figure 1A left). In contrast, 24 hours after surgery there was a significant increase in Evans Blue in the ipsilateral dorsal horn of nerve-injured animals as compared with sham controls (Figure 1A left). Evans Blue accumulation in the dorsal horn of nerve-injured animals was also significantly increased when the dye was administered 3 days after surgery. However, when Evans Blue was administered 7 days after surgery the dye accumulation in the dorsal horn of nerve-injured animals was not different from that of sham controls or naïve animals.

To independently assess the status of the BSCB we intravenously administered horse radishperoxidase (HRP) which, like Evans Blue bound to albumin, is not able to cross the normal intact BSCB. We found that HRP coats the lining of vessels but does not enter the parenchyma of the dorsal horn after intravenous injection in naïve animals (Figure 1B). However, when administered at 24 hours after CCI, HRP was readily visualized in the dorsal horn parenchyma. By quantifying HRP in the ipsilateral dorsal horn we found that the amount of HRP was significantly increased in the dorsal horn from nerve-injured animals as compared with that from control animals (Figure 1B). From the accumulation of HRP and of Evans Blue after nerve injury we conclude that the permeability of the BSCB increases after CCI. The increase in BSCB permeability is delayed by more than 6 hours and appears to peak about after 24 hours after the surgery. The increase in permeability is transient, with BSCB permeability returning to control levels by 7 days after CCI surgery.

#### Spared nerve injury (SNI) also causes increased BSCB permeability

To determine whether the increase in BSCB permeability was a unique consequence of CCI or whether increased permeability may be induced by other types of nerve injury, we examined the effects of axotomy of the sciatic nerve, in which the common peroneal and tibial nerves were transected. We found that 24 hours and 3 days after surgery Evans Blue accumulation in the dorsal horn was significantly increased in animals with SNI compared with that in animals with sham surgery or naïve control animals (Figure 1A right). There was no increase in Evans Blue accumulation 6 hours or 7 days after SNI. In addition, HRP was found to accumulate in the dorsal horn parenchyma when assessed at 24 hours after SNI (Figure 1B). Thus, we conclude that, like CCI, SNI causes a delayed and transient increase in BSCB permeability, and therefore that increased BSCB permeability may be a general consequence of injury to a peripheral nerve. Because the magnitude and time course of the increase in Evans Blue and HRP accumulation were similar for CCI and SNI we used only one nerve injury model, CCI, and one approach to assess BSCB permeability, Evans Blue administration, for the remainder of the present study.

#### CCI-induced increase in BSCB permeability is prevented by lidocaine

**Figure 2:**
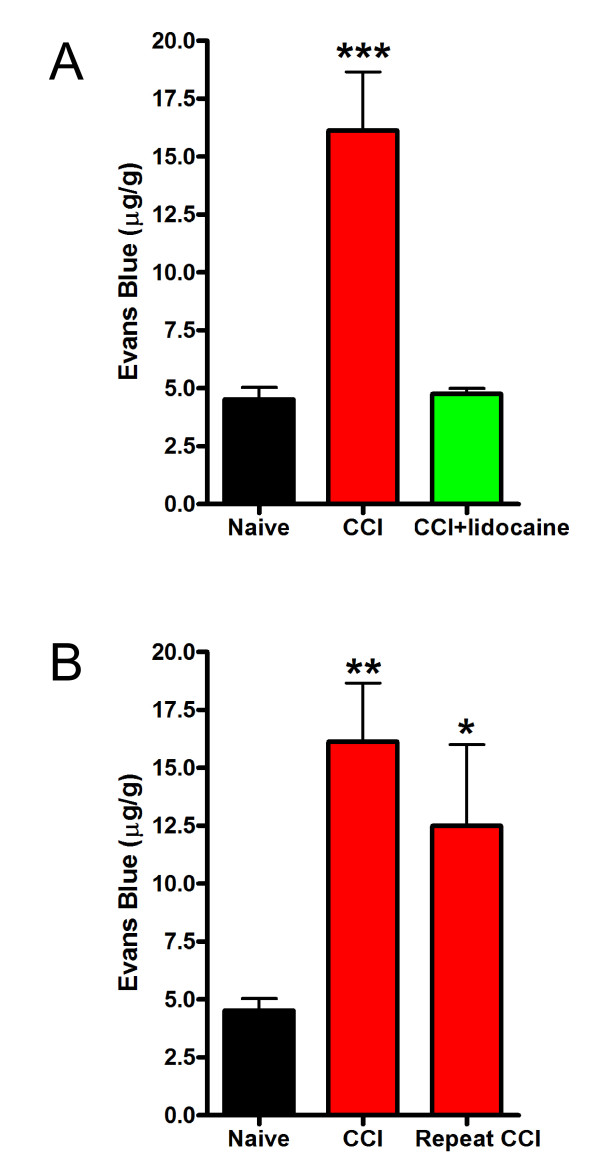
***The effect of local lidocaine block and sequential nerve injury on CCI-induced increase in BSCB permeability***. A: 2% of Lidocaine was applied to the sciatic nerve immediately following CCI, and Evans Blue extravasation in the ipsilateral lumbar spinal cord was measured 24 hours post CCI. B: A sequential CCI was performed on the right sciatic nerve after the initial CCI was performed in the left sciatic nerve 6 days previously. Evans blue extravasation was measured 7 days after initial CCI. All readings are from the left lumbar spinal cords (ipsilateral to the initial injury). Data are presented as mean ± SEM; *p < 0.05 **p < 0.01 compared to naïve; n = 5 per group.

To determine whether opening of the BSCB requires propagation of action potentials from the site of nerve injury, we investigated the effect of the local anaesthetic lidocaine. Lidocaine was applied directly to the nerves starting 30 min prior to injury to ensure that action potentials were blocked at the time of nerve transection. Gel foam soaked in lidocaine was placed around the nerves after transection and we administered Evans Blue 24 hours later. We found that administering lidocaine prevented the increase in Evans Blue accumulation after CCI (Figure 2A). From this finding we infer that action potential propagation in the transected nerves is required to cause the increase in BSCB opening after peripheral nerve injury.

#### Recovery of BSCB integrity after CCI due to loss of signal to open

The increase in BSCB permeability was transient with permeability returning to control levels by 7 days after nerve injury. We reasoned that the recovery of BSCB integrity might be due to loss of a drive that sustains BSCB opening or that the BSCB might become resistant to the driving signals. To differentiate between these possibilities we made a second nerve injury, on the side contralateral to the first injury, 6 days after the first injury. We found that there was a significant increase in Evans Blue accumulation in the dorsal spinal cord 24 hrs after the second nerve injury (Figure 2B). Thus, the BSCB was not resistant to opening when a drive to open was presented on day 6. Therefore, we conclude that the recovery of BSCB permeability was due to a recovery of the action potential-dependent drive to open after the nerve injury.

### Short-duration stimulation of C-fibers opens BSCB

#### C-fiber but not A-fiber stimulation causes increase in BSCB permeability

**Figure 3:**
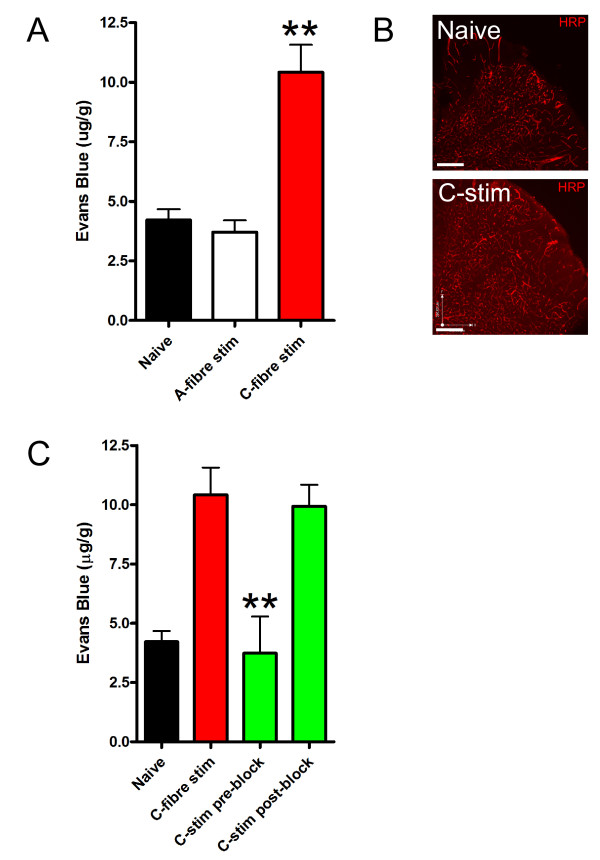
***C-fiber intensity stimulation increases BSCB permeability***. A: Sciatic nerve stimulation with C-fiber intensity, but not A-fiber intensity increases permeability to Evans Blue in rat spinal cord 24 hours post stimulation. Data are presented as mean ± SEM; **p < 0.01 compared to naïve, n = 4-8 per group. B: Representative spinal cord sections of HRP extravasation from naïve rats or rats 24 hours post C-fiber intensity stimulation of the sciatic nerve. C: C-fiber intensity stimulation-induced permeability is blocked by pre-treatment, but not post-treatment, with lidocaine. 2% lidocaine was applied to the sciatic nerve either 30 minutes before C-fiber stimulation (pre-block) or immediately after C-fiber stimulation (post-block). The duration of lidocaine treatment was 30 minutes in both cases. Data are presented as mean ± SEM; **p < 0.01 compared to C-fiber stimulation, n = 4-8 per group. All the readings are from lumbar spinal cord ipsilateral to nerve stimulation.

In order to determine which type of peripheral nerve fiber may drive opening of the BSCB, we made use of electrical stimulation of the intact sciatic nerve to differentially activate different classes of peripheral nerve fibers. As electrical stimulation for only several minutes in duration can produce peripheral vasodilation and plasma extravasation [20], and can induce long-lasting pain hypersensitivity [21], we examined effects of short-duration stimulation on Evans Blue accumulation in the ipsilateral dorsal spinal cord. We found that electrical stimulation that selectively activates A-fibers (1 mA; 10 Hz; 5 min) caused no change in Evans Blue accumulation (Figure 3A). By contrast, stimulation sufficient to recruit primary afferent C-fibers (10 mA; 10 Hz; 5 min) caused a delayed increase in Evans Blue accumulation: Evans Blue was not different from control 3 hrs after C-fiber stimulation (not illustrated), but was significantly increased 24 hrs after stimulation (Figure 3A). Electrical stimulation at C-fiber intensities also produced an increase in HRP in the ipsilateral dorsal spinal cord 24 hrs after the stimulation (Figure 3B). Thus, the short-duration electrical stimulation of C-fibers but not A-fibers was sufficient to cause a delayed increase in BSCB permeability.

#### Increase in BSCB permeability by C-fiber stimulation is blocked by lidocaine

It is conceivable that short duration C-fiber stimulation initiates long-lasting discharge in primary afferents and to investigate this possibility we applied lidocaine to the sciatic nerve either before or immediately after the electrical stimulation. We found that applying lidocaine prior to the stimulation prevented the increase in Evans Blue accumulation 24 hrs later (Figure 3C). In contrast, applying lidocaine immediately after the electrical stimulation had no effect on the Evans Blue accumulation at 24 hrs. Under the conditions used, blockade of action potential propagation develops within 15-20 minutes and therefore we conclude the C-fiber discharge during the electrical stimulation, and possibly in the immediate few minutes thereafter, acts as a trigger to cause the delayed increase in BSCB permeability.

#### Capsaicin applied to the sciatic nerve causes increased BSCB permeability at 24 hrs

**Figure 4:**
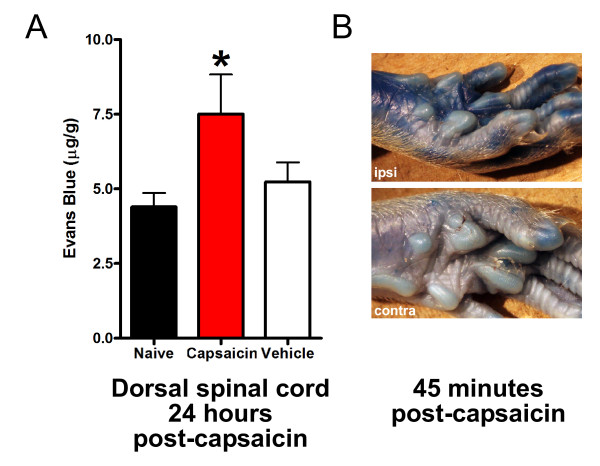
***Local application of capsaicin increases BSCB permeability***. A: 1% Capsaicin or vehicle was applied onto the sciatic nerve and Evans Blue extravasation was measured 24 hours later. All readings are from lumbar spinal cord ipsilateral to capsaicin or vehicle. Data are presented as mean ± SEM; **p < 0.01 compared to naïve, n = 5-9 per group. B: 1% Capsaicin was applied to the sciatic nerve and Evans Blue was injected immediately after capsaicin. Ipsilateral and contralateral paw images were captured 45 minutes after capsaicin treatment.

Neurogenic plasma extravasation is mediated through activating primary afferent C-fibers that express TRPV1 and are hence activated by capsaicin [9,22]. To determine whether activating this class of primary afferent might increase BSCB permeability we applied capsaicin to the sciatic nerve and measured Evans Blue in the dorsal spinal cord 24 hrs later. We found that capsaicin, but not vehicle control, caused an increase in Evans Blue at 24 hrs (Figure 4A). In contrast applying capsaicin to the sciatic nerve led to peripheral plasma extravasation that was apparent within 45 min (Figure 4B) Thus, we conclude that activating TRPV1-expressing C-fibers is sufficient to cause an increase in BSCB permeability.

### C-fiber stimulation and peripheral nerve injury cause delayed widespread opening of BSCB

#### Sciatic nerve C-fiber stimulation causes increase in BSCB permeability in spinal cord

**Figure 5:**
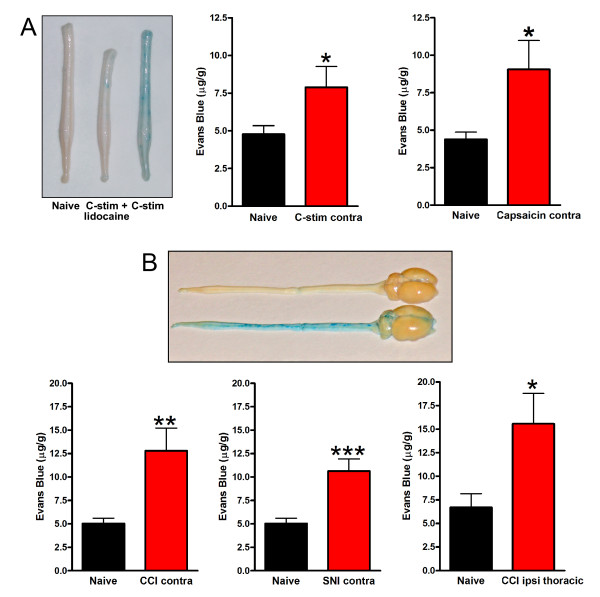
***Increased BSCB permeability extends throughout the spinal cord***. A: left panel, representative spinal cord from naïve rats, rats 24 hours post C-fiber stimulation or rats with lidocaine block before C-fiber stimulation. Middle and right panels, C-fiber stimulation (middle) or local application of capsaicin onto sciatic nerve (right) increases contralateral spinal cord Evans Blue permeability 24 hours post treatment. B: Increased permeability to Evans Blue in the lumbar spinal cord contralateral to CCI (left panel) or SNI (middle) and in thoracic spinal cord (right). Upper picture: representative spinal cord and brain from naïve rats, or rats 24 hours post PNI. Data are presented as mean ± SEM; *P < 0.05, **p < 0.01, **P < 0.001 compared to respective naïve groups, n = 6-9 per group.

In investigating the effects of electrical C-fiber stimulation we examined the contralateral as well as the ipsilateral lumbar dorsal spinal cord and found that the increase in Evans Blue accumulation 24 hrs after stimulation was produced in the contralateral, as well as the ipsilateral dorsal spinal cord (Figure 5A). Moreover the level of Evans Blue in the contralateral side was not different from on the side ipsilateral to the nerve stimulation. Increased Evans Blue accumulation in the contralateral dorsal cord was mimicked by applying capsaicin to the sciatic nerve (Figure 5A). The increases in Evans Blue accumulation in spinal cord was prevented by applying lidocaine to the sciatic nerve just prior to electrical stimulation (Figure 5A). Taking these findings together we conclude that the increase in BSCB permeability triggered by short-duration electrical stimulation of C-fibers was not restricted to the ipisilateral spinal dorsal horn but was widespread in the spinal cord.

#### PNI causes increase in BSCB and BBB permeability in thoracic and cervical spinal cord and in brain

We therefore questioned whether PNI might also cause widespread opening of the blood-CNS barriers. We found that Evans Blue accumulation was significantly increased throughout the spinal cord 24 hrs after CCI and SNI (Figure 5B). Thus, like C-fiber stimulation, injury to a peripheral nerve causes extensive opening of the BSCB. In addition, we measured Evans Blue accumulation in brain regions following CCI. Significant increases were found in caudal brain regions; brain stem Evans Blue levels were significantly increased at 3 days post CCI (p < 0.05). Progressively more rostral brain areas showed lower levels of Evans Blue accumulation; occipital cortex p = 0.058; cerebellum and frontal cortex showed no significant increase. Thus, in addition to increasing BSCB permeability, PNI causes disruption of the BBB in some regions of the brain.

## Discussion

Here we have discovered that peripheral nerve injury or electrical stimulation of C-fibers in the sciatic nerve produce opening of the blood-spinal cord and blood-brain barriers. The increase in BSCB permeability is prevented by applying lidocaine to the nerve prior to the nerve injury or electrical stimulation, indicating that action potential discharge is required. By contrast, applying lidocaine directly after the electrical stimulation had no effect on the subsequent increase in BSCB permeability. Increased BSCB permeability was also produced by direct application of capsaicin to the sciatic nerve. Taking our findings together, the most parsimonious explanation is that discharge activity in TRPV1-expressing C-fibers triggers a cascade of events which, after many hours, leads to an increase in BSCB and BBB permeability.  Our findings thus reveal a previously unknown function of TRPV1-expressing C-fibers.

This function of these afferents to cause opening of the BSCB was unanticipated as peripheral neurogenic plasma extravasation, which is also initiated by TRPV1-expressing C-fibers, develops rapidly upon electrical activation. Plasma extravasation has been found to begin within tens of seconds of the start of C fiber stimulation [20,23]. By contrast, BSCB permeability has been found to not increase during or within minutes after C fiber stimulation [13] or with intravenous administration of capsaicin [24]. Thus, the onset of the effect of TRPV1-expressing afferents on vascular permeability in peripheral tissues is dramatically different than that of the effect of these fibers on vascular permeability in the CNS. A second major difference between the effects of TRPV1-afferents on peripheral versus CNS vascular permeability is the duration of the increase: peripheral plasma extravasation ends within minutes of terminating electrical C-fiber stimulation [23], whereas the increase in BSCB permeability persists for days after the stimulation. A third difference is the localization of the increase in vascular permeability: plasma extravasation in the periphery is highly localized to the territory innervated by the nerve that is stimulated [25] whereas the increase in permeability in the CNS is evoked far beyond the anatomical distribution of the central terminals, occurring throughout the spinal cord. These major differences imply that, beyond being initiated by TRPV1-expressing C-fibers, the mechanisms for the sensory neuron-evoked increase in vascular permeability in the CNS are highly divergent from those producing plasma extravasation in the periphery.

Because the increase in BSCB permeability is delayed many hours after electrical C-fiber stimulation and the increase is not prevented by applying lidocaine to the nerve immediately after the stimulation, we infer that the activity evoked during the stimulation acts to trigger a cascade of events that culminates in the opening of the barrier. It is conceivable that the cascade may include transcription of critical genes and translation of the relevant gene products that act as mediators. In addition, or alternatively, to increased production of certain gene products, key elements of the BBB might become reduced, and in preliminary experiments we have found a decrease in the level of aquaporin-4, a component of the BBB, preceding the increase in permeability [19]. Given the widespread increase in vascular permeability in the CNS, a possible scenario may be that the cascade of events involves the production and release of a humoral mediator, or mediators, that act on the cellular and/or intercellular elements that maintain the intact barrier. Alternatively, there could be rostral spread of BSCB permeability from an initiation site in the lumbar dorsal horn or central release of diffusible mediator(s) that circulate within the cerebrospinal fluid. Or, the increased permeability might be caused by synaptically released mediators from neuronal pathways having widespread projections, such as those from brainstem or hypothalamic regions.

The increase in BSCB permeability might contribute to the development of pain hypersensitivity after PNI. Increased permeability may facilitate the entry of circulating cells such as monocytes [15] and T cells [17] that home to the dorsal horn near the site of termination of the central endings of injured primary afferents. Increased BSCB permeability could also allow entry of soluble factors that are normally excluded but that could contribute to pain hypersensitivity in the dorsal horn. For example, fibronectin is known to cross the opened BBB [26] and this is known to stimulate P2X4R expression in microglia [27], and this increase in P2X4 is critical for mechanical hypersensitivity after PNI [28,29]. Also, matrix metalloproteinases (MMPs) have been implicated in opening the BBB [30], and MMPs in the dorsal horn are critical for pain hypersensitivity after PNI [31,32]. However, the PNI-induced increase in BBB permeability is not sufficient on its own to cause pain hypersensitivity because permeability is increased throughout the spinal cord whereas pain hypersensitivity is typically restricted to the region near the nerve injury.

Our finding that the PNI-induced increase in BSCB permeability began within 24 hours and the permeability returning to the basal level by 7 days after PNI, is at earlier time points than a previous report using transection of the L4 spinal nerve [18]. It was found that L4 nerve injury caused an endogenous albumin accumulation at time points of one to ten weeks after the transection. The accumulation observed at those time points may reflect albumin that entered during the period of opening that we have discovered here. The recovery of BSCB integrity appears to be due to a gradual loss of the signals that are driving the opening, rather than to resistance of the BSCB to sustained signals to open, because we found here that the permeability increased upon a second nerve injury 7 days after the first. Thus, while the BSCB is only open for several days after nerve injury, repeated nerve injury may re-open the BSCB after it has recovered.

Traumatic and ischemic injuries to the CNS are well-known to cause localized disruption of the BBB which is considered critical to the pathophysiology [1,33,34]. With the stimuli used presently -- PNI, electrical C-fiber stimulation or capsaicin applied to the nerve -- there is no direct injury to areas where the BSCB and BBB permeability is increased. Thus, CNS injury cannot account for the increases in BSCB and BBB permeability we have found presently.

Our findings open up the possibility that PNI and activating TRPV1-expressing primary afferents may cause increased BSCB and BBB permeability in humans. If this is found to be the case, then there are several potential clinical implications of our findings. First, the BSCB and BBB would be much more permeable in situations of peripheral trauma, accidental or surgical, particularly when there is nerve damage. From this, one may expect that the pharmacokinetics of systemically administered drugs may be altered and the penetration into CNS might be enhanced. This enhanced drug penetration might increase the potency of agents where BBB permeability is limiting, but might also increase centrally-mediated side effects. A second potential clinical implication is that our findings may point to development of approaches to purposefully open the BBB in order to facilitate entry of drugs that normally have limited access to central targets. While it is unlikely that direct nerve injury would be used therapeutically, it is conceivable that short-duration C-fiber stimulation, done under general anaesthesia as in the studies here in rats, might be tolerated. However, by far the best approach would be through ascertaining the mediator or mediators involved in the afferent-induced opening of the BBB and to then develop treatments based on mimicking, in a safe way, the mechanism. A third possibility is that widespread opening of the BBB caused by PNI or activation of TRPV1-expressing C-fibers might contribute to the so-called 'sickness syndrome' [35] that follows injury. A final, and much more speculative, possibility to consider pertains to non-traumatic, non-ischemic disorders where disruption of the BBB appears critical for the pathophysiology, such as demyelinating, neuroimmune or neurodegenerative disorders. Our findings raise the possibility that in some such diseases activity of TRPV1-expressing C-fibers may contribute to the BBB disruption.

## Conclusions

In summary, we have discovered that peripheral nerve injury and activation of TRPV1-expressing C-fibers causes extensive opening of the BSCB and BBB. The onset of the opening is delayed by many hours after the stimulus and persists for several days. The opening of the BSCB may contribute to the pathophysiology of neuropathic pain hypersensitivity by creating a permissive environment for recruitment and infiltration of circulating immune cells into the spinal cord parenchyma. Given the widespread effect on vascular permeability in the CNS it is possible that activating the sensory nerves causes the release of a circulating factor, or factors, that causes breakdown of the barrier. Our findings have a number of potential clinical implications for situations where there is activation of TPRV1-expressing C-fibers, such as trauma or other noxious peripheral stimuli.

## Methods

### Animals

All animals were used in accordance with the guidelines of the Canadian Council on Animal Care. All protocols were approved by the Animal Care Committee of the Hospital for Sick Children. For all experiments male 250-300 g Sprague-Dawley (Charles River) were used.

### Peripheral Nerve injury models

Spared nerve injury (SNI) was performed as described previously [36]. Briefly, rats were anaesthetized by isofluorane inhalation and the left sciatic nerve exposed under aseptic conditions. The distal trifurcation of the sciatic nerve was identified and the tibial and common peroneal branches ligated and cut, leaving the sural branch intact. The wound was sutured closed and the animals allowed to recover and returned to their housing. Chronic constriction injury (CCI) was performed as described previously [37]. A polyethylene cuff (PE-60, 2 mm in length) was placed around the exposed left sciatic nerve and the wound closed as above. Sham surgeries, exposure of the sciatic nerve only, were also performed. To assess the effect of sequential nerve injury on the permeability of the BSCB, CCI was performed on the left sciatic nerve followed by a further CCI on the right nerve 7 days later.

### Electrical stimulation of sciatic nerve

The sciatic nerve was isolated as described above and a bipolar stimulating hook electrode was used for electrical stimulation. The stimulation parameters for C-fiber strength stimulation were: 5 min train duration, 500 μsec stimulus pulse duration, 10 Hz, 10 mA. The stimulation parameters for A-fiber strength stimulation were: 5 min train duration, 150 μsec stimulus pulse duration, 10 Hz, 1 mA.

### Lidocaine block

0.1 ml of 2% lidocaine (Novocol Pharmaceutical, Canada) was applied topically to the sciatic nerve for 30 minutes before nerve injury or C-fiber stimulation. The efficacy of local lidocaine block was confirmed by lack of peripheral plasma extravasations 45 minutes after C-fiber stimulation.

### Capsaicin stimulation

Capsaicin (1% in Ethanol: Tween 80: saline 1:1:8, Sigma-Aldrich) or vehicle was applied to the sciatic nerve.

### Evans Blue assay

Blood spinal cord permeability was determined by Evans Blue extravasation into the spinal cord. Evan's blue dye (2%, 4 ml/kg) was infused through the jugular vein of anaesthetized rats. After 45 minutes animals were perfused with PBS. The spinal cord and supraspinal tissues were immediately dissected and dura mater removed. The lumbar spinal cord (L4-6), and middle thoracic cord were further dissected. Tissue was incubated in 600 μl of formamide (Sigma-Aldrich) at 60C for 72 h. Evan's Blue concentration was then determind by spectrophotometry at 620 nm.

### Horseradish peroxidise assay

BSCB permeability was further assayed using a horseradish peroxidise (HRP) assay. HRP (3 mg/kg) was infused through the jugular vein of anaesthetized animals. 45 minutes later animals were perfused transcardially with 10% formalin solution. Following post-fixation and sucrose cryoprotection, spinal cord sections (50 μm) were cut on a microtome. Sections were incubated in cy3-labelled tyramide solution (Perkin Elmer) for 7 minutes to measure HRP activity. Resulting fluorescence images were captured and integrated pixel density measured using Volocity software.

### Statistics

Evan's Blue concentration was assessed across all groups using one way ANOVA and Newman-Keuls post-hoc multiple comparisons. Where appropriate, naïve and experimental groups were compared using *t*-tests.

## Competing interests

The authors declare that they have no competing interests.

## Authors' contributions

SB participated in the design of the study, carried out experiments, analyzed data and wrote the paper. XJL carried out experiments, analyzed data and participated in writing of the paper. CK designed and carried out experiments, and analyzed data. MWS conceived of the study, participated in its design and coordination, and wrote the paper. All authors read and approved the text.
